# Production of Four ^15^N-Labelled Cobalamins via Biosynthesis Using *Propionibacterium freudenreichii*

**DOI:** 10.3389/fmicb.2021.713321

**Published:** 2021-08-13

**Authors:** Mengle Wang, Stefan Asam, Jianqi Chen, Matthias Ehrmann, Michael Rychlik

**Affiliations:** ^1^Chair of Analytical Food Chemistry, Technical University of Munich, Freising, Germany; ^2^Chair of Technical Microbiology, Technical University of Munich, Freising, Germany

**Keywords:** biosynthesis, *Propionibacterium freudenreichii*, chemically defined medium, vitamin B12, cobalamins, ^15^N-labelling

## Abstract

Cobalamins (vitamin B12) are required by humans for their essential roles as enzyme cofactors in diverse metabolic processes. The four most common cobalamin vitamers are hydroxocobalamin (OHCbl), adenosylcobalamin (AdoCbl), methylcobalamin (MeCbl), and cyanocobalamin (CNCbl). Humans are not able to synthesise cobalamins *de novo* and thus must acquire them from external sources. Therefore, a reliable and robust analytical method to determine the cobalamins in dietary sources is highly required. For such a purpose, stable isotope dilution assays (SIDAs) with LC-MS/MS are most suited due to their superior sensitivity, specificity, and ability to compensate for matrix effects and analyte loss during sample work-up. However, a critical bottleneck for developing a SIDA method for cobalamins is the availability of stable isotope-labelled internal standards. In the present study, we harnessed the potential of *Propionibacterium (P.) freudenreichii* for the biosynthesis of ^15^N-labelled cobalamins. First, we developed a chemically defined medium (CDM) containing ammonium sulphate as a single nitrogen source except three essential vitamins that supported long-term stable growth of *P. freudenreichii* throughout continuous transfers. The CDM was further optimised for cobalamin production under different incubation schemes. With the optimised CDM and incubation scheme, fully ^15^N-labelled cobalamins were obtained in *P. freudenreichii* with a final yield of 312 ± 29 μg/L and 635 ± 102 μg/L, respectively, for [^15^N]-OHCbl and [^15^N]-AdoCbl. Additionally, an optimised incubation process under anaerobic conditions was successfully employed to produce specifically labelled [^15^N, ^14^N_2_]-cobalamins, with a yield of 96 ± 18 μg/L and 990 ± 210 μg/L, respectively, for [^15^N, ^14^N_2_]-OHCbl and [^15^N, ^14^N_2_]-AdoCbl. The labelled substances were isolated and purified by solid phase extraction and semi-preparative HPLC. Chemical modifications were carried out to produce [^15^N]-CNCbl and [^15^N]-MeCbl. Eventually, ^15^N-labelled compounds were obtained for the four cobalamin vitamers in high chromatographic and isotopic purity with desired ^15^N-enrichment and labelling patterns, which are perfectly suited for future use in SIDAs or other applications that require isotopologues.

## Introduction

**FIGURE 1:**
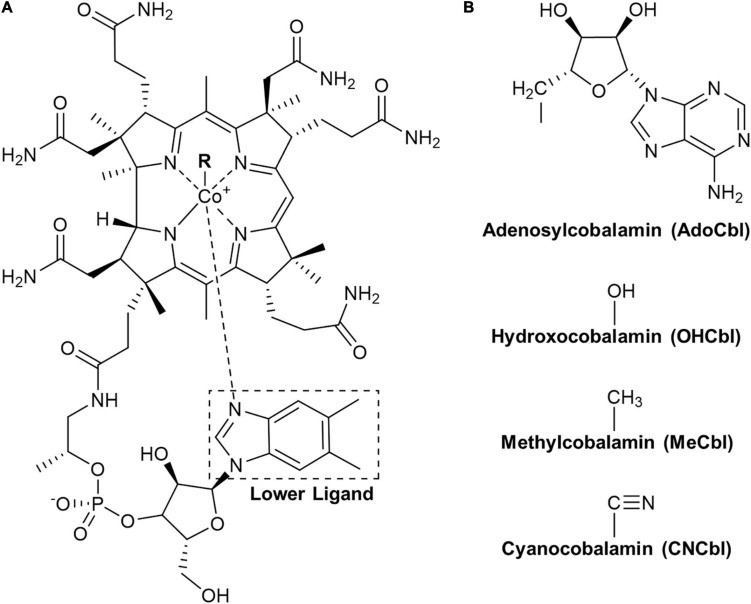
The cobalamin structure. **(A)** Core structure of the cobalamin with the lower ligand 5,6-dimethylbenzimidazole (DMB) indicated by the box. **(B)** Structures of upper ligands (R) for individual vitamers with corresponding names and abbreviations given below.

Cobalamin, commonly known as vitamin B12 (B12), has one of the most complex structure of all vitamins, which is composed of a cobalt central atom coordinated with a tetracyclic corrin system, an upper β-ligand (cyano, methyl, hydroxyl, or adenosyl group) and 5,6-dimethylbenzimidazole (DMB) as the lower α-ligand (Figure 1). In human physiology, methylcobalamin (MeCbl) and adenosylcobalamin (AdoCbl) are required as cofactors for the metabolic functions of two enzymes: methionine synthase (E.C. 2.1.1.13) and methylmalonyl-CoA mutase (E.C. 5.4.99.2) (Kolhouse and Allen, 1977). Hydroxocobalamin (OHCbl) and cyanocobalamin (CNCbl) can be converted into these two forms by human metabolism after absorption. The two mentioned enzymes play vital roles in various metabolic reactions involved in DNA synthesis, branched-chain amino acid and odd-chain fatty acid metabolisms (Stabler, 1999). In nature, cobalamins are exclusively produced by certain bacteria and archaea (Roth et al., 1996). In human nutrition, the major dietary sources for cobalamins are animal-derived foods due to the natural food-chain enrichment. Plant-derived foods are considered to be devoid of cobalamins unless fermented with certain bacteria. Inadequate intake or malabsorption results in cobalamin deficiency, affecting the normal functions of the blood and nervous system (Green et al., 2017). To prevent cobalamin deficiency, one of the most straightforward approach is monitoring the dietary intake to meet the recommended dietary allowance (RDA) of 2.4 μg/day for adults and 0.9 to 1.8 μg/day for children (Green and Miller, 2013). For such a purpose, robust, reliable, and sensitive analytical methods for the determination of cobalamins in known and potential cobalamin-containing food sources are highly required.

Common analytical methods for cobalamins include microbiological assays (MBA) (Kelleher and Broin, 1991), high performance liquid chromatography with UV detection (HPLC-UV) (Heudi et al., 2006; Marley, 2009; Guggisberg et al., 2012; Chamlagain et al., 2015) and liquid chromatography coupled to tandem mass spectrometry (LC-MS/MS) (Szterk et al., 2012; Zironi et al., 2013). The reported methods suffer from different drawbacks: (i) lack of sensitivity requiring high sample amounts for food analysis, (ii) lack of specificity to distinguish cobalamins from structural analogues with vitamin activity (vitamers) and other inactive corrinoids, and (iii) lack of accuracy due to the low recoveries caused by matrix interference, especially in MS measurements. Therefore, it has become common practice to determine cobalamins not in their natural forms but as CNCbl after treating the sample with cyanide. Some of the mentioned analytical challenges can be improved by this technique, but only by accepting the loss of information about the natural compositions of cobalamins in the food sample. However, optimising the LC-MS/MS technique is another option, as this technique already is sufficiently sensitive and specific. Deficiencies in accuracy can be solved by using stable isotope dilution assays (SIDAs). With the application of stable isotope-labelled internal standards, losses during sample work-up and matrix effects during MS measurements are completely compensated due to the identical physical and chemical properties of the analyte and their corresponding isotopologues (Asam and Rychlik, 2015). To date, SIDA has not been applied to native cobalamins in food samples.

The critical bottleneck for developing a SIDA method for cobalamins is the availability of stable isotope-labelled internal standards. The chemical synthesis of cobalamin, with more than 70 synthesis steps, is extremely complicated and expensive (Woodward, 1973; Eschenmoser and Wintner, 1977). Therefore, the industrial production of cobalamins exclusively relies on microbial production (Martens et al., 2002). Researchers in this field often combine biological and chemical approaches to prepare cobalamin-related compounds, with the complex corrin ring structure obtained from biological sources (Friedrich et al., 1960; Stupperich et al., 1988; Fonseca and Escalante-Semerena, 2001; Allen and Stabler, 2008; Crofts et al., 2013; Widner et al., 2017). Regarding the preparation of labelled cobalamins, current publications were mainly based on the following two approaches: (i) isotopes were introduced via chemical synthesis in the lower ligand structure before supplementation to model bacteria for the guided biosynthesis of the labelled target compound (Carkeet et al., 2006; Allen and Stabler, 2008) or (ii) labelled upper ligands (e.g., labelled cyanide ^13^C^15^N^–^) were used for reaction with co(I)balamin, a reduced form, to produce the final labelled compound (Kolhouse et al., 1991; D’Ulivo et al., 2017). However, the chemical synthesis of isotopically labelled lower ligands can be complex, time consuming, and expensive. Moreover, guided biosynthesis does not always guarantee a unified labelling in the final product. Labelling the upper ligand is practical when a limited number of labels are needed, but not sufficient for labelling cobalamins for the purpose of SIDA. In order to avoid mass spectrometric overlaps between analytes and labelled standards, a sufficient mass shift is required for the labelled standard.

Among all efficient cobalamin producers, *Propionibacterium (P.) freudenreichii* is one of the most common species used to produce cobalamins in industrial scale (Martens et al., 2002). It is also preferred due to its generally recognised as safe (GRAS) and qualified presumption of safety (QPS) status. Moreover, *P. freudenreichii* is a hardy bacterium that has low nutritional requirements and can survive and adapt to various environments (Thierry et al., 2011). *P. freudenreichii* is prototrophic for all amino acids and nucleotides and is able to grow, under anaerobic conditions, in a minimal media containing ammonium as the sole nitrogen source, if a carbon source, minerals and two to four vitamins (pantothenate, biotin, thiamin, and p-aminobenzoic acid) are supplied (Falentin et al., 2010).

Thus, the preparation of labelled cobalamins seems possible by fermentation of *P. freudenreichii* in a chemically defined medium (CDM) and replacing the sole nitrogen source with ^15^N. Based on the reported biosynthetic pathway of cobalamins (Fang et al., 2017), ^15^N may be introduced stepwise starting from the first precursor L-glutamate via C5 pathway into the final product via *de novo* synthesis, as *P. freudenreichii* is self-sufficient for this amino acid.

Therefore, the aim of our study was: (i) to develop a chemical defined medium that allows biosynthetic preparation of ^15^N-labelled cobalamins by *P. freudenreichii* in high isotopic purity, (ii) isolation of the biosynthesised compounds as pure substances, and (iii) structural characterisation of the labelled compounds to check their suitability as potential internal standards in SIDAs.

## Materials and Methods

### Strains, Chemicals, and Solvents

*Propionibacterium freudenreichii* subsp. *freudenreichii* DSM 20271^T^ (DSM 20271) was obtained from Deutsche Sammlung von Mikroorganismen und Zellkulturen GmbH (DSMZ, Braunschweig, Germany) and was kept as cryopreserved stock culture at −80°C in glycerol.

All chemicals were obtained from Sigma-Aldrich (Steinheim, Germany) unless otherwise specified. Tween 80 was purchased from GERBU Biotechnik GmbH (Heidelberg, Germany). [^15^N_2_]-ammonium sulphate [(^15^NH_4_)_2_SO_4_, 99%] was purchased from Eurisotop (Saarbrücken, Germany). Tryptone, yeast extract and agar were purchased from Roth (Karlsruhe, Germany). HPLC-UV grade solvents were obtained from VWR (Ismaning, Germany). LC-MS grade solvents were from Honeywell (Seelze, Germany).

### Growth Media and General Incubation Conditions

#### Complex Media

Sodium lactate broth (SLB) contained 10 g of tryptone, 5 g of yeast extract and 16.7 g of sodium DL-lactate syrup (60%, w/w) per litre. The pH of the medium was adjusted to 7.0 using 4 M NaOH before autoclaving at 118°C for 10 min. For the preparation of sodium lactate agar (SLA), 15 g of agar per litre was additionally added to form the solid medium.

#### Chemically Defined Media (CDM)

The composition of the CDM is given in Table 1. The pH was adjusted to 7.0 by 4 M NaOH and the medium was sterilised via filtration (CytoOne Bottle Top Filtration Unit, 0.2 μm, PES, Starlab GmbH, Hamburg, Germany). For CDM fortified with glucose (CDMG), 10 g/L of glucose was further added while preparing the medium. For the preparation of ^15^N-labelled CDMG ([^15^N]-CDMG), (^15^NH_4_)_2_SO_4_ was substituted for unlabelled (NH_4_)_2_SO_4_.

**TABLE 1:** Composition of chemically defined medium developed in the current study.

**Components**	**Concentration (g/L)**
Sodium DL-lactate*	12
(NH_4_)_2_SO_4_	3
K_2_HPO_4_	8.7
KH_2_PO_4_	6.8
MgSO_4_.7H_2_O	0.2
FeSO_4_.7H_2_O	0.01
MnSO_4_.H_2_O	0.02
ZnSO_4_.H_2_O	0.01
NaCl	0.2
CaCl_2_.2H_2_O	0.132
CoCl_2_.6H_2_O	0.002
Calcium pantothenate	0.001
Biotin	0.001
Thiamin.HCl	0.001
Sodium acetate trihydrate	6
Sodium pyruvate	1
α-ketoglutaric acid	1
Succinic acid	1
Myo-inositol	0.1
Tween 80	0.5

*20 g of sodium DL-lactate syrup (60%, w/w) used.

#### Incubation Conditions

All cultures were grown at 30°C. Semi-anaerobic incubation was performed statically in tightly closed screw-cap Schott bottles or centrifuge tubes under normal atmosphere. Aerobic incubation was carried out in Erlenmeyer flasks under shaking conditions (120 rpm). Anaerobic incubation was performed inside a Bactron anaerobic chamber (Sheldon Manufacturing Inc., Cornelius, OR, United States) statically.

### Cell Activation and Preculture Preparation

The frozen stock culture was thawed and streaked onto SLA for activation. The inoculated agar plates were incubated in a sealed jar with a generated anaerobic atmosphere (AnaeroGen, Thermo Fisher Scientific, Madison, WI, United States) for 3–5 days. Subsequently, five single colonies from the plates were transferred into 14 mL SLB and incubated semi-anaerobically for 3 days. The bacteria were further propagated in 14 mL SLB (2% inoculum, v/v) for two more generations under the same incubation conditions before transferring to the CDM or CDMG.

### Growth Observation in CDM

To assess the growth of *P. freudenreichii* in CDM, 14 mL CDM inoculated with 2% (v/v) of the preculture grown in SLB described above was incubated semi-anaerobically for 5 days. If apparent sedimentation of cells were observed in the bottom of the centrifuge tube, next propagation was carried out by transferring 2% (v/v) of the culture after mixing to a fresh 14 mL CDM for next generation. Purity of the cultures were checked periodically by streaking out onto the SLA.

### *In vivo* Cobalamin Biosynthesis Under Different Incubation Schemes

*Propionibacterium freudenreichii* was grown in CDM or CDMG under different cultivation schemes (Table 2) for the optimisation of *in vivo* cobalamin biosynthesis. The cultivations were performed in 250 mL Schott bottles for the semi-anaerobic phase and were transferred to 500 mL Erlenmeyer flasks with cotton plugs for the aerobic phase when applied. For each cultivation, 250 mL of medium was used. The inocula (2%, v/v) were 4th generation culture grown in CDM. For each scheme, two biological replicate cultures were used.

**TABLE 2:** Conditions of different cultivation schemes.

**Scheme**	**Medium**	**Culture conditions**
a	CDM	7-day semi-anaerobic
b	CDM	14-day semi-anaerobic
c	CDM	7-day semi-anaerobic 1-day aerobic
d	CDM	7-day semi-anaerobic 2-day aerobic
e	CDM	7-day semi-anaerobic 3-day aerobic
f	CDM	7-day semi-anaerobic 7-day aerobic
g	CDM + 10 μM DMB	14-day semi-anaerobic
h	CDM	7-d semi-anaerobic 10 μM DMB added* 3-day aerobic
i	CDMG	5-day semi-anaerobic
j	CDMG	5-day semi-anaerobic 3-day aerobic
k	CDMG	5-day semi-anaerobic NaHCO_3_ (1 M, 20 mL) added* Glucose (250 g/L, 15 mL) added* 3-day aerobic

*Added after the semi-anaerobic phase.

### Production of Fully and Partially ^15^N-Labelled Cobalamins

#### Two-Phase Incubation for Production of ^15^N-Cobalamins in [^15^N]-CDMG (Full Labelling)

For biosynthesis of fully ^15^N-labelled cobalamins, *P. freudenreichii* was grown in [^15^N]-CDMG. After activation in SLB, the strain was sub-cultured in 14 mL [^15^N]-CDMG (2% inoculum, v/v) by serial transfer every 5 days to minimise carry-over of complex media. The cultures were maintained under semi-anaerobic conditions. Afterward, 250 mL media was inoculated with 5 mL (2% inoculum, v/v) of a 5-day old preculture (4th generation in [^15^N]-CDMG) for a larger-scale batch fermentation. The inoculated batch culture was first incubated semi-anaerobically for 5 days and then incubated aerobically with shaking (120 rpm) for another 3 days. Before the aerobic incubation period, the culture was neutralised with 20 mL of 1 M NaHCO_3_ solution and further supplemented with 15 mL of 250 g/L glucose stock solution.

#### Production of [^15^N, ^14^N_2_-DMB]-Cobalamin in DMB-Supplemented [^15^N]-CDMG Under Anaerobic Conditions (Specific Partial Labelling)

To produce partially labelled cobalamins with unlabelled lower ligand, [^15^N]-CDMG supplemented with 10 mg/L DMB was used as the fermentation broth. 200 mL of DMB-supplemented [^15^N]-CDMG was employed in a 250 mL high pressure glass bottle (Schott, Mainz, Germany) for each cultivation. The bottles were sealed gas tight by butyl rubber lids.

Before inoculation, the media were purged with a stream of argon for 2.5 h using two sterile cannulas. The flow of argon was introduced via a long cannula reaching below the liquid level to thoroughly displace air inside the media while stirring. The other cannula was placed above the liquid level as a gas outlet. The gas composition of the headspace was monitored by a gas analyser (PA 7.0, Witt-Gasetechnik, Witten, Germany). After 2.5 h of constant gassing, the oxygen was no longer detected in the headspace. Then, the gas flow was stopped, and the bottles were simultaneously locked gas tight.

Inoculation was performed inside the anaerobic chamber to minimise oxygen exposure. Media were inoculated with 4 mL of a 5-day old preculture semi-anaerobically propagated in 14 mL of [^15^N]-CDMG (4th generation). Afterward, a static incubation was carried out inside the anaerobic chamber for 5 days.

### Cell Harvesting

The cells were harvested by centrifugation (10,000 × *g*) at 4°C for 20 min and washed once with phosphate buffer saline (PBS, pH = 7.4). Afterward, cell pellets were stored at −20°C until further use.

### Extraction and Purification of Bacterial Cells

Wet bacterial cells (200 mg for analytical purposes and 400 mg for preparative purposes) were weighed into a 25 mL extraction vial and was mixed with 10 mL of extraction buffer (50 mM sodium acetate buffer, pH = 4.0). The mixture was thoroughly vortexed and further stirred with magnetic stir bar for 20 min at room temperature. Afterward, the homogenate was heated in a boiling water bath for 30 min and cooled immediately in an ice-water bath. The sample was further incubated at 37°C for 1 h in a light-protected shaking water bath. After cooling down in the ice-water bath, the sample mixture was transferred to a 50 mL centrifuge tube. The residue in the extraction vial was washed twice each with 3 mL of extraction buffer and then transferred into the centrifuge tube. The sample was centrifuged for 30 min (4°C and 3,220 × *g*). The supernatant was collected for solid phase extraction (SPE) using C18 columns (500 mg, 6 mL, Discovery^R^ DSC-18, Supelco, Bellefonte, PA, United States). The columns were first activated with 6 mL of methanol and equilibrated with 6 mL of extraction buffer before a complete loading of the sample solutions. After washing the columns again with 2 mL of extraction buffer, an elution step was carried out using 3 mL of MeOH/H_2_O (70/30; v/v). The eluant was evaporated to dryness under a stream of nitrogen at 40°C and stored at −20°C until further use.

### Further Preparation of ^15^N-Labelled Cobalamin Standards

#### Preparation of [^15^N]-AdoCbl

The cells from [^15^N]-CDMG were extracted as described in section “Extraction and Purification of Bacterial Cells.” [^15^N]-AdoCbl was isolated by semi-preparative HPLC as described below.

#### Preparation of [^15^N]-CNCbl

After extraction of cells and clean-up of the extracts from [^15^N]-CDMG incubation as described in section “Extraction and Purification of Bacterial Cells,” the eluent was exposed to ambient light for 40 min before adding 30 μL of NaCN solution (1% in water, w/v). The mixture was left to react at room temperature protected from light for 2 h. The solution was manually shaken every 30 min to ensure complete mixing. Afterward, the mixture was dried at 40°C under a stream of nitrogen and stored at −20°C before further purification. After reconstitution in water, the produced [^15^N]-CNCbl was isolated from the reaction solution by collecting corresponding peaks from semi-preparative HPLC as described below.

#### Preparation of [^15^N]-MeCbl

The reduction and methylation procedures were adopted from Brown et al. (1984) with some changes. A portion of isolated pure [^15^N]-AdoCbl was reconstituted in 1 mL of distilled water and photolysed to [^15^N]-OHCbl by exposure to ambient light for 40 min. The solution was then degassed with argon for 1 h in a sealed glass vial. Subsequently, a freshly prepared NaBH_4_ solution (25 mg/mL, 200 μL) in NaOH (0.1 M, pre-purged with argon) was added dropwise to the solution through a septum and the reaction was left to proceed at room temperature under argon for 30 min. Afterward, methyl iodide (50 μL) was added to the reaction vial, and the mixture was left to react at room temperature under argon for 30 min. Acetone (200 μL) was added to quench residue NaBH_4_ in the solution. The mixture was then dried at 40°C under a stream of nitrogen and stored at −20°C before further purification. After reconstitution in water, the produced [^15^N]-MeCbl was isolated from the reaction solution by collecting corresponding peaks from semi-preparative HPLC as described below.

#### Preparation of [^15^N, ^14^N_2_-DMB]-OHCbl

The cells obtained from DMB-supplemented [^15^N]-CDMG were extracted and purified as previously described in section “Extraction and Purification of Bacterial Cells.” After the elution step of SPE, the sample solution was exposed to ambient light for 40 min with thorough vortexing every 10 min. The light treated eluant was then dried at 40°C under nitrogen and reconstituted in 300 μL of water for further purification. The partially labelled [^15^N, ^14^N_2_]-OHCbl was isolated by collecting corresponding peaks from semi-preparative HPLC as described below.

In addition, we also isolated fully ^15^N-labelled [^15^N]-OHCbl from cells obtained from [^15^N]-CDMG incubation. The cells were extracted as described in section “Extraction and Purification of Bacterial Cells” and [^15^N]-OHCbl was isolated by collecting corresponding peaks from semi-preparative HPLC as described below.

### Analytical and Semi-Preparative HPLC-DAD

HPLC-DAD was performed on a Shimadzu HPLC system (Shimadzu, Kyoto, Japan) consisting of an auto-sampler (SIL-20A), a liquid chromatograph (LC-20AD) and a diode array detector (SPD-M20A). A YMC Triart C18 column (150 × 3.0 mm, 3 μm, Dinslaken, Germany) was used for chromatographic separation at 30°C. Two different linear gradients based on different combinations of mobile phases were used for the analytical and preparative purposes, respectively.

For the analysis of cobalamins and cobalamin precursors in bacterial cultures during method development and optimisation, 50 mM ammonium acetate buffer (pH = 4) and methanol were used as mobile phases A and B, respectively. The gradient started at 10% B and linearly increased to 40% B in the first 22 min. The gradient was held at 40% B for 2 min before it was raised to 95% within 2 min. After it maintained at 95% B for 2 min, the gradient returned to 10% B within 2 min and the column was equilibrated for 10 min before next injection. The flow rate was 0.3 mL/min and the injection volume was 20 μL. The cellular concentrations of OHCbl and AdoCbl were determined by external calibration based on the calibration graphs in the Supplementary Figures 8, 9).

For the preparation of ^15^N-labelled cobalamins, chromatographic separation was performed using 25 mM sodium acetate buffer (pH = 4) and methanol as mobile phases A and B, respectively. The gradient also started at 10% B and increased to 40% B in the first 30 min. Afterward, the gradient followed the same path as for the analytical method. However, a different flow rate of 0.45 mL/min was used for preparative purposes.

### LC-MS/MS

Liquid chromatography coupled to tandem mass spectrometry analyses were performed with a Shimadzu Nexera X2 UHPLC-LCMS 8050 triple quadrupole mass spectrometer under positive electrospray ionisation (ESI) mode. Briefly, samples were injected into a Hydrosphere C18 column (150 × 30 mm, 3 μm, YMC, Dinslaken, Germany) at 30°C. The mobile phases A and B were 0.1% acetic acid in water and pure methanol, respectively. The detailed LC and MS parameters are summarised in the Supplementary Material.

### UHPLC-Q-TOF-MS

UHPLC-Q-TOF-MS analyses were performed on a Waters Acquity UHPLC System (Waters, Eschborn, Germany) coupled to a Bruker maXis UHR-ToF-MS with an Apollo II ESI source (Bruker Daltonics, Bremen, Germany). A YMC Triart C18 column (150 × 3.0 mm, 3 μm, YMC, Dinslaken, Germany) was used for separation at 30°C. 10 mM ammonium formate buffer (pH = 4) and methanol were used as mobile phases A and B, respectively. The detailed operating parameters are shown in the Supplementary Material.

## Results and Discussion

### Development of Chemically Defined Medium for Stable Growth of *P. freudenreichii*

For the preliminary experiments, a minimal defined medium (medium M, Supplementary Table 2) was employed on the basis of defined media previously reported for the growth of *P. freudenreichii*, with some modifications (Glatz and Anderson, 1988; Dherbécourt et al., 2008). This medium met the reported minimum nutritional requirements of *P. freudenreichii* (Vorobjeva, 1999; Falentin et al., 2010), as it contained sodium lactate as the carbon source, ammonium sulphate as the sole nitrogen source, mineral salts and eight vitamins. Amino acids and nucleotides were not included as they were proven to be non-essential for *P. freudenreichii* to grow.

When inoculated from SLB (2% inoculum, v/v), the *P. freudenreichii* was able to grow in medium M for two generations but failed to continue after the 3rd transfer. However, we aimed to develop a CDM that supports long-term stable growth of *P. freudenreichii* with ammonium as sole nitrogen source throughout consistent transfers, as the CDM was intended for later ^15^N-labelling experiments. Serial transfers contribute to decreasing carry-over of non-labelled compounds from the preculture complex medium, i.e., SLB, leading to a better isotopic purity of the final products. The medium M, therefore, was not adequate for this purpose. To improve the growth, organic acids from TCA cycle were added to the medium as their stimulatory effects have been previously reported (Ferguson et al., 1978). These organic acids were non-nitrogenous and therefore did not introduce additional nitrogen into the medium. However, non-essential vitamins were omitted to further remove nitrogen-containing nutrients other than ammonium sulphate. Eventually, a stable and continuous growth of *P. freudenreichii* over 10 generations was observed in the final CDM listed in Table 1. The developed CDM was further tested for cobalamin production.

### *In vivo* Cobalamin Biosynthesis Under Different Incubation Schemes

#### Cobalamin Precursors Formed in *P. freudenreichii* Grown in the CDM

**FIGURE 2:**
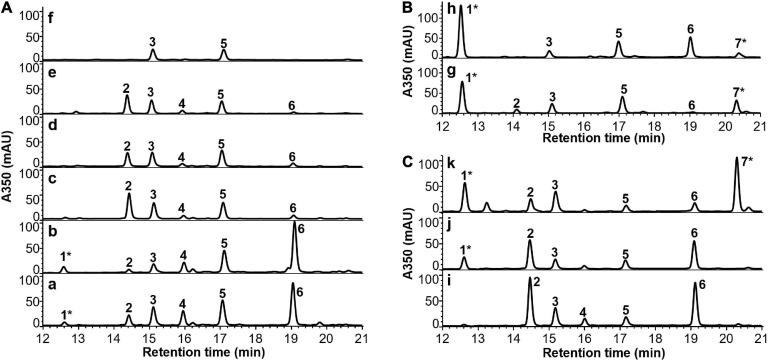
HPLC-DAD chromatograms of cell extracts of *P. freudenreichii* grown in CDM or CDMG under different cultivation schemes indicated on the upper left corner (details in Table 2). The identities of the numbered peaks were determined by comparing to the standards of OHCbl (1) and AdoCbl (7) or by UHPLC-QTOF-MS in terms of AdoCbi-GDP (2), FAD (3), AdoCbi-P (4), FMN (5) and AdoCbi (6) (see Supplementary Figures 1–6). **(A)** *P. freudenreichii* grown in CDM following cultivation schemes a–f. **(B)** *P. freudenreichii* grown in CDM with the addition of DMB before and after semi-anaerobic incubation following cultivation schemes g and h, respectively. **(C)** *P. freudenreichii* grown in CDMG following cultivation schemes i–k. Asterisk* indicates intact cobalamin structures.

In order to evaluate if the developed CDM can further support *in vivo* cobalamin biosynthesis, *P. freudenreichii* was grown under different cultivation schemes (Table 2) in the CDM, and cells were extracted and analysed by HPLC-DAD. The cultivation schemes were designed based on the reported biosynthetic pathway of cobalamins in *P. freudenreichii*, which is divided into two phases: (i) anaerobic/semi-anaerobic phase for the synthesis of cobalamin precursors from δ-aminolevulinic acid (ALA) until formation of 5′-deoxyadenosylcobinamide-GDP (AdoCbi-GDP) and (ii) aerobic phase that requires molecular oxygen for the conversion of flavin mononucleotide (FMN) into the lower ligand DMB before attachment to the corrin ring via a nucleotide loop. The metabolite profiles of cells from different batch cultures are summarised in Figure 2. The identities of relevant metabolite peaks were determined based on UHPLC-QTOF-MS measurements and UV-Vis spectra (Supplementary Figures 1–5).

HPLC-DAD analysis demonstrated that mainly cobalamin precursors were present in cell extracts when *P. freudenreichii* was grown in CDM under semi-anaerobic conditions up to 14 days. The most abundant precursor detected was 5′-deoxyadenosylcobinamide (AdoCbi). Corresponding peaks from 5′-deoxyadenosylcobinamide-phosphate (AdoCbi-P) and AdoCbi-GDP were also observed, but in lower intensities. Detectable levels of OHCbl were found in cells from semi-anaerobic incubation phase, which is to be expected as the fermentation was not conducted under strictly anaerobic conditions. When a second aerobic phase was employed during cultivation, the intensity of AdoCbi-GDP increased while the peak of AdoCbi diminished. Unexpectedly, the synthesised AdoCbi-GDP was accumulated in the cells without being further converted into AdoCbl when incubated aerobically up to 3 days. Moreover, the cobalamin precursors were completely depleted in the cells after an extended aerobic incubation period of 7 days. Interestingly, FMN and flavin adenine dinucleotide (FAD), the direct and indirect precursors of DMB biosynthesis (Taga et al., 2007), were found in all cell extracts from different cultivation schemes. These results suggest that the incomplete cobalamin biosynthesis was not caused by a lack of available precursors in the cells grown in the CDM.

#### Complete Cobalamins Synthesised in *P. freudenreichii* Grown in the CDM With DMB Supplementation

**FIGURE 3:**
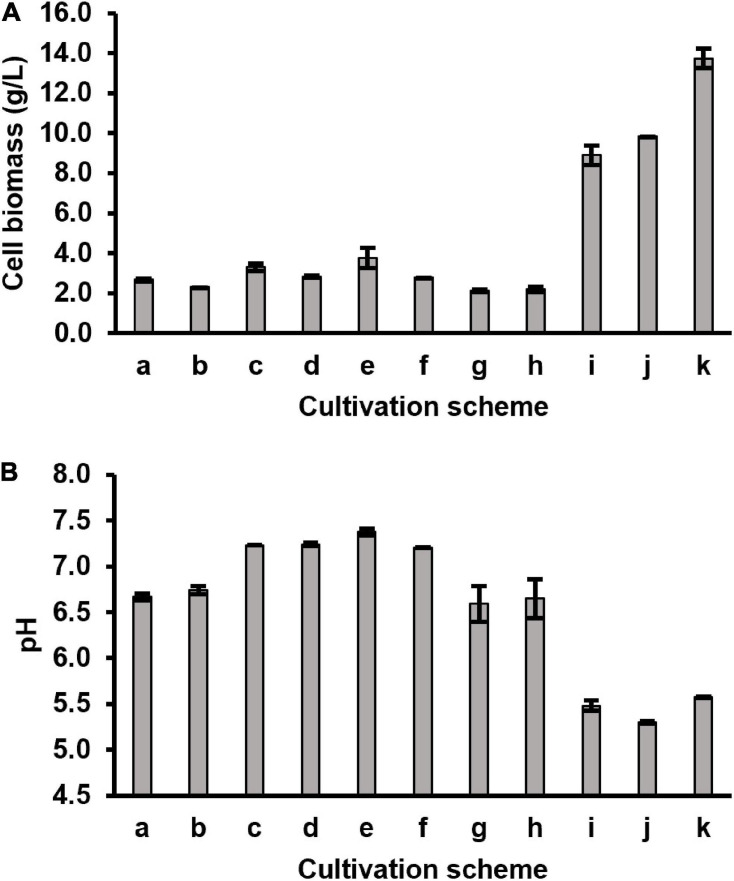
Harvested cell biomass **(A)** and pH **(B)** of the spent broth from different cultivation schemes. Two biological replicates for each scheme (*n* = 2).

To investigate if DMB was the limiting precursor for the further conversion of AdoCbi-GDP to AdoCbl, *P. freudenreichii* was cultured in the CDM supplemented with DMB under semi-anaerobic conditions for 14 days in two ways. 10 μM of DMB was either added to CDM at the beginning of the incubation or first supplied to the culture after 7 days of semi-anaerobic incubation. HPLC-DAD analysis showed that AdoCbl and OHCbl were present in cell extracts from both cultivations (Figure 2B), indicating that *P. freudenreichii* was able to incorporate provided DMB to form AdoCbl when grown in the CDM. Therefore, it appeared to us that the *in vivo* DMB synthesis was not effective in *P. freudenreichii* incubated in the CDM, resulting in an insufficient supply of lower ligand needed for the final step of cobalamin synthesis. The reasons for the ineffective DMB synthesis, however, remain to be elucidated. Surprisingly, cell biomasses and pH of the DMB-supplemented cultures were similar to the values of the non-supplemented ones after 14-day semi-anaerobic incubation (Figure 3). These results suggest that the growth of *P. freudenreichii* in CDM was not enhanced when intact cobalamins were synthesised inside the cells.

#### Enhanced Cell Growth and Completed Cobalamin Synthesis in CDMG

Although CDM could support stable growth of *P. freudenreichii* for generations, low cell biomasses were obtained during all batch cultivations using CDM with or without DMB supplementation (Figure 3). The pH values of the cultured media did not drop below 6.5 (originally pH 7.0), indicating that the limited cell growth could be due to exhaustion of carbon sources rather than to inhibition by produced acids. Therefore, glucose (10 g/L in final media) was added as extra carbon source to CDM (CDMG) to check if the growth of *P. freudenreichii* can be improved. After 5-day of semi-anaerobic incubation in CDMG (scheme i in Table 2), cell biomass increased from 2.7 g/L (7-day semi-anaerobic incubation, scheme a in Table 2) to 8.9 g/L, with pH value of the culture dropping to 5.5 (Figure 3). The addition of glucose, therefore, significantly enhanced the cell growth. HPLC-DAD analysis showed that the CDMG cell extracts from semi-anaerobic phase contained both AdoCbi-GDP and AdoCbi in abundant amounts (Figure 2C). With an additional 3-day aerobic incubation, OHCbl was observed in the cells while AdoCbi-GDP and AdoCbi were still present in high levels (Figure 2C).

As previously reported, the optimum pH for *P. freudenreichii* to grow is between pH 6 and 7 (Hsu and Yang, 1991; Ye et al., 1996; Vorobjeva, 1999) and growth decreases distinctly at pH values below 5.5 and ceases below pH 5 (Hettinga and Reinbold, 1972). To maintain the culture pH around 7, periodical neutralisation of fermented acids by alkaline agents is a common practice during industrial B12 production using *P. freudenreichii* (Martens et al., 2002). Therefore, we introduced a neutralisation step using 20 mL of 1 M NaHCO_3_ after 5-day semi-anaerobic incubation. Afterward, additional glucose (3.75 g) was added to the culture for providing more energy for cell growth and cobalamin synthesis before the 3-day aerobic phase. By doing so, the cell biomass further increased to 13.7 g/L (Figure 3A) and the majority of precursors were converted into intact cobalamins in harvested cells (Figure 2C). The culture pH afterward dropped to 5.6.

### Anaerobic Incubation for Specific Partial Labelling

#### The Necessity of Using Partially ^15^N-Labelled OHCbl for SIDA

OHCbl exists not only as a natural cobalamin but also can be the degradation product from other naturally occurring forms. It has to be noted that this degradation is inevitable when handling cobalamins. Therefore, if fully ^15^N-labelled OHCbl is used as internal standard for the analysis of OHCbl in future LC-MS/MS applications, the unavoidable generation of fully ^15^N-labelled OHCbl from other fully labelled internal standards will cause severe errors on the accurate quantification of OHCbl. Therefore, it is necessary to use a labelled internal standard that can be clearly distinguished by LC-MS/MS in a so-called dual isotope design from the fully labelled degradation product. One of the easiest approaches to solve this issue is to use specifically ^15^N-labelled OHCbl with an unlabelled lower ligand as internal standard. For such a reason, we further optimised the cultivation scheme to produce specifically labelled [^15^N, ^14^N_2_-DMB]-OHCbl, even though we were able to prepare fully ^15^N-labelled OHCbl from cells grown in the [^15^N]-CDMG cultivation.

#### *P. freudenreichii* Ceased Growth in Strict Anaerobic Conditions Without DMB Supplementation

To obtain partially labelled cobalamins in high purity, the procedures for biosynthesis must be modified for *P. freudenreichii* to use unlabelled DMB from the media rather than synthesising the labelled one *in vivo*. As the conversion of FMN to DMB requires molecular oxygen, incubation of *P. freudenreichii* in strictly anaerobic condition would be an effective way to block the *in vivo* DMB synthesis. Therefore, we tried to grow *P. freudenreichii* in CDMG under strictly anaerobic conditions without DMB supplementation. However, we observed that the growth was rather limited and even ceased after three generations. Under semi-anaerobic conditions, the presence of trace oxygen supported the growth of *P. freudenreichii* in CDM and CDMG, but only low amounts of cobalamins were synthesised and cobalamin precursors were the major forms in the cultures (Figure 2). Under strictly anaerobic condition without provided DMB, the synthesis of intact cobalamins is obstructed. It may be assumed that this is the reason for the decreased growth. When the cobalamin carry-over from previous generations is depleted, the growth ceases. Supplementing DMB is therefore necessary for reasonable production of cobalamins. However, supplementing DMB into the inoculum media for enhancing cell growth under anaerobic incubation is not ideal. The unlabelled nitrogen from DMB might be introduced into the corrin ring via unknown metabolic pathways, resulting in non-unified labelling of final compounds in the later batch cultivations. In a comprised way, *P. freudenreichii* was sub-cultured in CDMG under semi-anaerobic conditions first to ensure a good growth in the inoculum before inoculating into batch media of CDMG supplemented with DMB for anaerobic cultivation. Special care was taken to guarantee strict anaerobic conditions. The batch media was previously purged with argon and stored in the anaerobic chamber to reduce dissolved oxygen. Inoculation and incubation were performed in the anaerobic chamber to minimise oxygen exposure. Furthermore, the incubation time was kept as short as 5 days to minimise the possibility of DMB involved in other pathways than being incorporated into the cobalamins as lower ligand.

The cell biomass from the batch fermentation was 11.7 g/L with pH of medium dropping to 5.6. This revised cultivation scheme was eventually adopted to produce the partially labelled cobalamins using DMB-supplemented [^15^N]-CDMG.

### Yields of ^15^N-Labelled Cobalamins

*P. freudenreichii* was grown in [^15^N]-CDMG to produce fully ^15^N-labelled cobalamins following the previously developed procedures. The intracellular yields of [^15^N]-OHCbl and [^15^N]-AdoCbl in the harvested cells were 24.0 ± 2.0 μg/g (*n* = 3) and 48.7 ± 5.9 μg/g (*n* = 3), respectively. The corresponding volumetric yields of [^15^N]-OHCbl and [^15^N]-AdoCbl were 312 ± 29 μg/L and 635 ± 102 μg/L, respectively. Regarding the production of partially ^15^N-labelled cobalamins ([^15^N, ^14^N_2_-DMB]-cobalamins), the cells grown in the DMB-supplemented [^15^N]-CDMG contained 7.4 ± 1.5 μg/g (*n* = 2) of [^15^N, ^14^N_2_]-OHCbl and 77 ± 15 μg/g (*n* = 2) of [^15^N, ^14^N_2_]-AdoCbl. The corresponding volumetric yields were 96 ± 18 μg/L and 990 ± 210 μg/L, respectively, for [^15^N, ^14^N_2_]-OHCbl and [^15^N, ^14^N_2_]-AdoCbl.

The obtained yields are comparable to the results of Hugenschmidt et al. (2010), who reported a B12 yield of 900 μg/L for the same strain (DSM 20271) grown in a complex medium based on whey permeate with cobalt and DMB supplementation. According to Hugenschmidt et al. (2010), the DSM 20271 was not among the best B12 producing strains as a maximum B12 production of 2.5 mg/L was obtained among 37 natural *P. freudenreichii* strains investigated. Therefore, the production of ^15^N-labelled cobalamins can be further improved by using more capable *P. freudenreichii* strains in the future. Continuously controlling culture pH over the fermentation period might be another effective approach with respect to improving B12 yields as Quesada-Chanto et al. (1998) obtained significantly higher B12 yields of 2.7 mg/L for the DSM 20271 with automatic pH adjustment to 6.5 during the whole fermentation period.

### Isolation and Characterisation of Labelled Compounds

The biosynthesised ^15^N-labelled cobalamins were kept in the harvested cells at −20°C for long-term storage due to the low chemical stability of cobalamins. A portion of the cells was extracted for further preparation of the labelled compounds. Isolation of the labelled cobalamins from bacterial cells after SPE clean-up and further chemical modifications were performed using semi-preparative HPLC.

**FIGURE 4:**
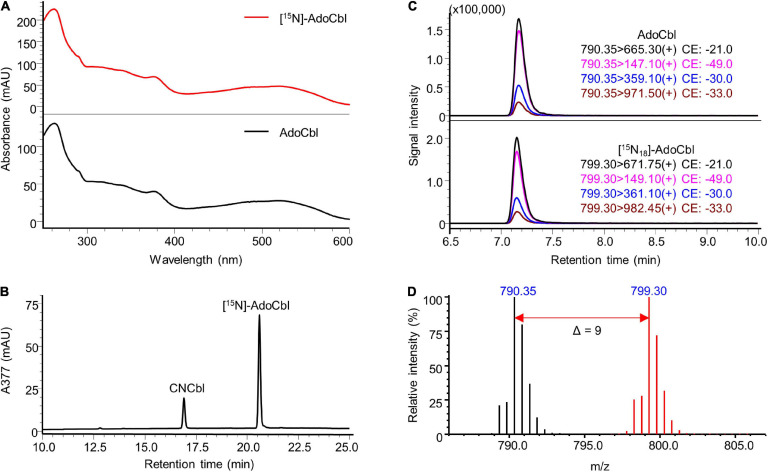
Characterisation of prepared [^15^N]-AdoCbl. **(A)** UV-Vis spectrum of the prepared [^15^N]-AdoCbl in comparison to that of the AdoCbl standard. **(B)** HPLC-DAD analysis at a wavelength of 377 nm of the prepared substance showed a major peak at the same retention time of unlabelled AdoCbl standard (see Supplementary Figure 6). CNCbl shown on the chromatogram was added as internal standard to determine the absolute concentration of [^15^N]-AdoCbl. **(C)** LC-MS/MS analysis of [^15^N]-AdoCbl monitoring mass transitions corresponding to [^15^N_18_]-AdoCbl (lower trace). The LC-MS/MS chromatogram of unlabelled AdoCbl is shown as a reference (upper trace). **(D)** Overlaid mass spectra of [^15^N]-labelled AdoCbl (red) and unlabelled AdoCbl (black). The mass shift in [M + 2H]^2+^ values indicated by the red arrow confirmed the labelling of 18 nitrogen atoms. MS spectra were acquired in selected ion monitoring (SIM) mode with a m/z interval of 0.5.

**FIGURE 5:**
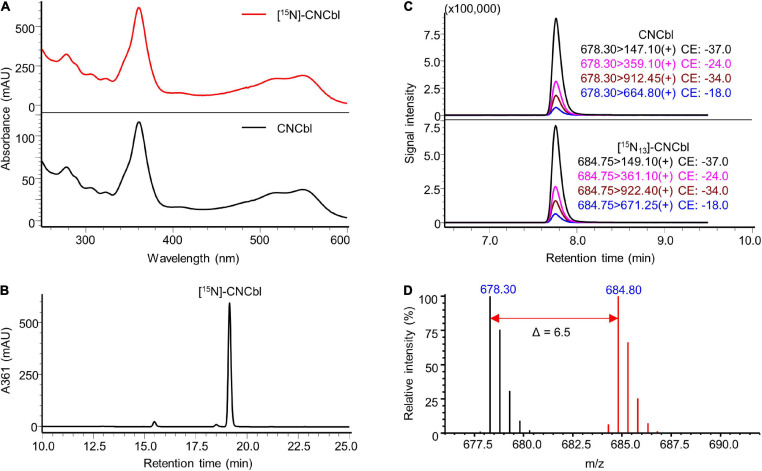
Characterisation of prepared [^15^N]-CNCbl. **(A)** UV-Vis spectrum of the prepared [^15^N]-CNCbl in comparison to that of the CNCbl standard. **(B)** Preparative HPLC-DAD at a wavelength of 361 nm of the product from cyanidation reaction showed a major peak of [^15^N]-CNCbl, which has an identical retention time to that of the CNCbl standard under the same elution conditions (see Supplementary Figure 7). **(C)** LC-MS/MS analysis of [^15^N]-CNCbl monitoring mass transitions corresponding to [^15^N_13_]-CNCbl (lower trace). The LC-MS/MS chromatogram of unlabelled CNCbl is shown as a reference (upper trace). **(D)** Overlaid mass spectra of [^15^N]-labelled CNCbl (red) and unlabelled CNCbl (black). The mass shift in [M + 2H]^2+^ values indicated by the red arrow confirmed the labelling of 13 nitrogen atoms. MS spectra were acquired in selected ion monitoring (SIM) mode with a m/z interval of 0.5.

**FIGURE 6:**
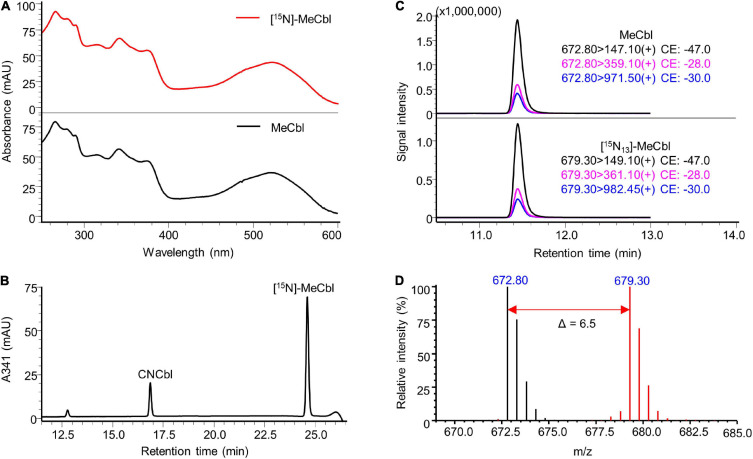
Characterisation of prepared [^15^N]-MeCbl. **(A)** UV-Vis spectrum of the prepared [^15^N]-MeCbl in comparison to that of the MeCbl standard. **(B)** HPLC-DAD analysis at a wavelength of 341 nm of the product from methylation reaction showed a major peak of [^15^N]-MeCbl at the same retention time of unlabelled MeCbl standard (see Supplementary Figure 6). **(C)** LC-MS/MS analysis of [^15^N]-MeCbl monitoring mass transitions corresponding to [^15^N_13_]-MeCbl (lower trace). The LC-MS/MS chromatogram of unlabelled MeCbl is shown as a reference (upper trace). **(D)** Overlaid mass spectra of [^15^N]-labelled MeCbl (red) and unlabelled MeCbl (black). The mass shift in [M + 2H]^2+^ values indicated by the red arrow confirmed the labelling of 13 nitrogen atoms. MS spectra were acquired in selected ion monitoring (SIM) mode with a m/z interval of 0.5.

The identity of the prepared labelled compounds was confirmed by HPLC-DAD and LC-MS/MS in comparison to respective unlabelled standards. The characterisation of [^15^N]-AdoCbl is summarised in Figure 4. The UV-Vis spectrum of the isolated [^15^N]-AdoCbl was identical to that of the unlabelled AdoCbl (Figure 4A). The HPLC-DAD chromatogram of [^15^N]-AdoCbl showed a single major peak eluting at the same time as the unlabelled AdoCbl (Figure 4B and Supplementary Figure 6), indicating a high chromatographic purity. Mass spectrometric results confirmed that the majority of [^15^N]-AdoCbl was fully labelled [^15^N_18_]-AdoCbl, as the base peak shifted from m/z 790.30 ([M + 2H]^2+^) to m/z 799.30 (Δ m/z = + 9 in double charged forms). As expected, there was no spectral overlap between labelled and unlabelled compounds. Moreover, unlabelled AdoCbl was not at all detected in the [^15^N]-labelled product, proving that no carry-over from the inoculum took place, which might also have been an issue for mass spectrometric interferences (Figure 4D). A peak correspondingx to [^15^N_18_]-AdoCbl, which eluted at the same retention time of unlabelled AdoCbl, was observed when analysed by LC-MS/MS in the multiple reaction monitoring (MRM) mode (Figure 4C). The intensity ratios between different labelled mass transitions from [^15^N_18_]-AdoCbl were comparable to those of unlabelled transitions from AdoCbl, thus meeting an important quality control criterion in tandem mass spectrometry for unequivocal identification. Taken together, the labelled compound was obtained in high purity and is perfectly suited for future use in SIDA or other applications that require isotopologues. Similar results were obtained for [^15^N]-CNCbl and [^15^N]-MeCbl (Figures 5, 6).

**FIGURE 7:**
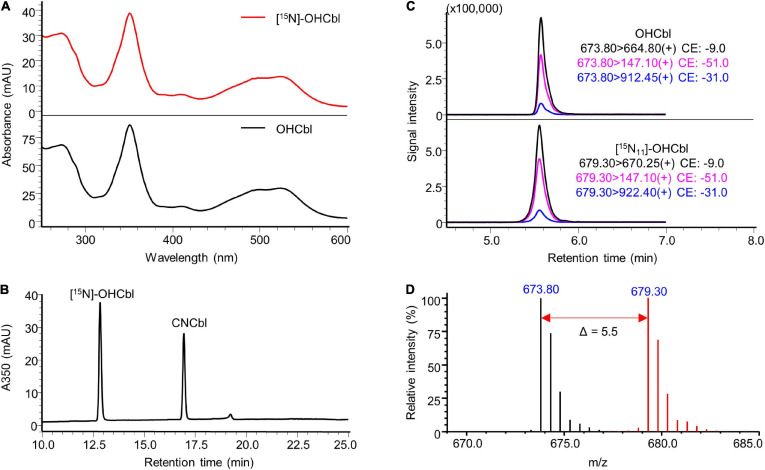
Characterisation of prepared [^15^N, ^14^N_2_-DMB]-OHCbl. **(A)** UV-Vis spectrum of the prepared [^15^N, ^14^N_2_-DMB]-OHCbl in comparison to that of the OHCbl standard. **(B)** HPLC-DAD analysis at a wavelength of 350 nm of the prepared substance showed a major peak at the same retention time of unlabelled OHCbl standard (see Supplementary Figure 6). CNCbl shown on the chromatogram was added as internal standard to determine the absolute concentration of [^15^N, ^14^N_2_-DMB]-OHCbl. **(C)** LC-MS/MS analysis of [^15^N, ^14^N_2_-DMB]-OHCbl monitoring mass transitions corresponding to [^15^N_11_, ^14^N_2_-DMB]-OHCbl (lower trace). The LC-MS/MS chromatogram of unlabelled OHCbl is shown as a reference (upper trace). **(D)** Overlaid mass spectra of [^15^N, ^14^N_2_-DMB]-OHCbl (red) and unlabelled OHCbl (black). The mass shift in [M + 2H]^2+^ values indicated by the red arrow confirmed the labelling of 11 nitrogen atoms. MS spectra were acquired in selected ion monitoring (SIM) mode with a m/z interval of 0.5.

In terms of [^15^N, ^14^N_2_-DMB]-OHCbl prepared from DMB-supplemented [^15^N]-CDMG under anaerobic conditions, the base peak shifted from m/z 673.80 ([M + 2H]^2+^) to m/z 679.30 (Δ m/z = + 5.5 in double charged forms) in the LC-MS spectra (Figure 7), indicating a fully ^15^N-labelled corrin ring and the existence of two unlabelled nitrogen atoms in the lower ligand DMB. Residues of unlabelled OHCbl were not detected in the labelled compound. The corresponding peak of [^15^N_11_, ^14^N_2_-DMB]-OHCbl in the LC-MS/MS chromatogram further confirmed the anticipated labelling. The synthesised [^15^N, ^14^N_2_-DMB]-OHCbl had sufficient mass increment that does not overlap with natural isotopologues of OHCbl (Figure 7D). Taken together, the modification of the biosynthetic strategy yielded specifically labelled [^15^N, ^14^N_2_]-OHCbl with the desired labelling pattern that allows the differentiation from fully labelled [^15^N]-OHCbl as degradation product from other labelled cobalamins.

## Conclusion

We successfully prepared four ^15^N-labelled cobalamin vitamers via a biosynthetic approach utilising *P. freudenreichii* in combination with chemical modifications. First, we developed a CDM containing ammonium sulphate as the sole nitrogen source that supports stable growth of *P. freudenreichii*. The CDM was further optimised together with the incubation process for a successful *in vivo* cobalamin biosynthesis. The optimised CDM (CDMG) and incubation process were applied to produce ^15^N-labelled cobalamins by substituting (NH_4_)_2_SO_4_ with (^15^NH_4_)_2_SO_4_ in the medium. Fully ^15^N-labelled [^15^N]-AdoCbl was isolated directly from cell extracts by semi-preparative HPLC. [^15^N]-CNCbl and [^15^N]-MeCbl were prepared with further cyanidation and methylation reactions, respectively, using isolated [^15^N]-AdoCbl or [^15^N]-OHCbl from cell extracts. Specifically labelled [^15^N, ^14^N_2_-DMB]-OHCbl was successfully obtained from *P. freudenreichii* cells grown in DMB-supplemented [^15^N]-CDMG with an optimised incubation process under anaerobic conditions. After semi-preparative HPLC purification, all four labelled compounds demonstrated high HPLC and MS purity with expected ^15^N-enrichment. The developed biosynthetic production of ^15^N-labelled cobalamins is simple, effective, and economical. Future improvement on B12 yield is possible by further optimisation on process control, such as static control of culture pH, and by selecting *P. freudenreichii* strains that have better B12 producing capability.

## Data Availability Statement

The raw data supporting the conclusions of this article will be made available by the authors, without undue reservation.

## Author Contributions

MW, ME, and MR conceptualised the research and designed the experiments. MW developed the CDM and analytical methods, performed all microbiological experiments, characterised labelled compounds, and wrote the manuscript. MW and JC performed extractions and purifications of bacterial cells, and were responsible for all preparative HPLC experiments. SA, ME, and MR revised the manuscript. All authors contributed to data analysis and interpretation.

## Conflict of Interest

The authors declare that the research was conducted in the absence of any commercial or financial relationships that could be construed as a potential conflict of interest.

## Publisher’s Note

All claims expressed in this article are solely those of the authors and do not necessarily represent those of their affiliated organizations, or those of the publisher, the editors and the reviewers. Any product that may be evaluated in this article, or claim that may be made by its manufacturer, is not guaranteed or endorsed by the publisher.
