# Supplementary material for: Production of Four 15N-Labelled Cobalamins via Biosynthesis Using Propionibacterium freudenreichii
Source: Front Microbiol. 2021 Aug 13;12:713321. doi: 10.3389/fmicb.2021.713321 (PMC8414983; doi:10.3389/fmicb.2021.713321)
Supplement: Supplementary file 1 [file Table_1.DOCX]

Supplementary Material

# LC-MS/MS

A linear gradient was used for elution as follows: 0-2 min, 10% B; 2-3 min, increase to 30% B; 3-11 min, increase to 50% B; 11-12.5 min, 50% B; 12.5-14 min, increase to 99% B; 14-15 min, 99% B; 15-17 min, return to 10% B; 17-27 min, 10% B. The flow rate was 0.3 mL/min. The MS source parameters were set as follows: ESI (+) mode, interface voltage of 4 kV, interface temperature of 340 °C, heat block temperature of 400 °C, desolvation line temperature of 90 °C, nebulizing gas flow of 3 L/min, heating gas flow of 10 L/min, drying gas flow of 10 L/min. Collision-induced dissociation (CID) gas pressure was set at 310 kPa from 0 to 9 min and changed to 270 kPa from 9 min until the end of the run. The detailed MRM parameters are summarized in Table S1.

# UHPLC-Q-TOF-MS

A linear gradient was used for separation as follows: 10 min pre-run time, 10% B; 0-20 min, increase to 40% B; 20-22 min, 40% B; 22-26 min, increase to 95% B; 26-28 min, 95% B; 28-30 min, return to 10%. The flow rate was 0.3 mL/min. The MS source parameters were set as follows: ESI (+) mode, capillary voltage of 4.5 kV, end plate offset of 500 V, dry heater of 200 ºC, nitrogen flow rate of 10 L/min, and nebulizer pressure of 2.0 bar. Data acquisition was performed in data dependent acquisition (DDA) mode with fragmentation of the three most abundant peaks per scan at a mass range from m/z 50 to m/z 1500. The injection volume was 5 µL in partial loop mode. The mass spectrometer was calibrated by injecting ESI-L Low Concentration Tuning Mix (Agilent Technologies, Santa Clara, CA, USA). Internal calibration of the spectra was performed by injecting ESI-L Low Concentration Tuning Mix (1:4 diluted in acetonitrile), introduced by a switching valve.

# List of supplementary tables and figures:

**Table S1.** MRM parameters of LC-MS/MS for unlabelled and labelled cobalamins.

**Table S2.** Composition of the minimum defined medium (medium M).

**Figure S1.** UV-Vis spectrum (A) and UHPLC-QTOF-MS analysis (B) of the peak #2 (AdoCbi-GDP) on Figure 2 with the proposed fragmentation pattern (C).

**Figure S2.** UV-Vis spectrum (A) and UHPLC-QTOF-MS analysis (B) of the peak #4 (AdoCbi-P) on Figure 2 with the proposed fragmentation pattern (C).

**Figure S3.** UV-Vis spectrum (A) and UHPLC-QTOF-MS analysis (B) of the peak #6 (AdoCbi) on Figure 2 with the proposed fragmentation pattern (C).

**Figure S4.** UV-Vis spectrum (A) and UHPLC-QTOF-MS analysis (B) of the peak #3 (FAD) on Figure 2 with the proposed fragmentation pattern (C).

**Figure S5.** UV-Vis spectrum (A) and UHPLC-QTOF-MS analysis (B) of the peak #5 (FMN) on Figure 2 with the proposed fragmentation pattern (C).

**Figure S6.** HPLC-DAD chromatogram of a standard solution composed of four cobalamin standards under analytical conditions.

**Figure S7.** HPLC-DAD chromatogram of a standard solution composed of seven standards under semi-preparative conditions.

**Figure S8.** Calibration curve of OHCbl for HPLC-DAD.

**Figure S9.** Calibration curve of AdoCbl for HPLC-DAD.

**Table S1.** MRM parameters of LC-MS/MS for unlabelled and labelled cobalamins.

| **Analyte** | **Precursor ion m/z** | **Product ion m/z** | **Q1 Pre-bias (V)** | **CE (V)** | **Q3 Pre-bias (V)** | **Dwell time (ms)** |
| --- | --- | --- | --- | --- | --- | --- |
| OHCbl | 673.80 | 664.80 | 36.0 | 9.0 | 20.0 | 70.0 |
|  |  | 147.10 | 20.0 | 51.0 | 28.0 | 70.0 |
|  |  | 912.45 | 20.0 | 31.0 | 28.0 | 70.0 |
| [^15^N_11_, ^14^N_2_-DMB]-OHCbl | 679.30 | 670.25 | 36.0 | 9.0 | 20.0 | 70.0 |
|  |  | 147.10 | 20.0 | 51.0 | 28.0 | 70.0 |
|  |  | 922.40 | 20.0 | 31.0 | 28.0 | 70.0 |
| AdoCbl | 790.35 | 665.30 | 22.0 | 21.0 | 26.0 | 70.0 |
|  |  | 147.10 | 22.0 | 49.0 | 26.0 | 70.0 |
|  |  | 359.10 | 22.0 | 30.0 | 26.0 | 70.0 |
|  |  | 971.50 | 24.0 | 33.0 | 38.0 | 70.0 |
| [^15^N_18_]-AdoCbl | 799.30 | 671.75 | 22.0 | 21.0 | 26.0 | 70.0 |
|  |  | 149.10 | 22.0 | 49.0 | 26.0 | 70.0 |
|  |  | 361.10 | 22.0 | 30.0 | 26.0 | 70.0 |
|  |  | 982.45 | 24.0 | 33.0 | 38.0 | 70.0 |
| CNCbl | 678.30 | 147.10 | 34.0 | 37.0 | 28.0 | 70.0 |
|  |  | 359.10 | 34.0 | 24.0 | 14.0 | 70.0 |
|  |  | 664.80 | 20.0 | 18.0 | 20.0 | 70.0 |
|  |  | 912.45 | 20.0 | 34.0 | 28.0 | 70.0 |
| [^15^N_13_]-CNCbl | 684.75 | 149.10 | 34.0 | 37.0 | 28.0 | 70.0 |
|  |  | 361.10 | 34.0 | 24.0 | 14.0 | 70.0 |
|  |  | 671.25 | 20.0 | 18.0 | 20.0 | 70.0 |
|  |  | 922.40 | 20.0 | 34.0 | 28.0 | 70.0 |
| MeCbl | 672.80 | 147.10 | 20.0 | 47.0 | 28.0 | 100.0 |
|  |  | 359.10 | 36.0 | 28.0 | 26.0 | 100.0 |
|  |  | 971.50 | 20.0 | 30.0 | 38.0 | 100.0 |
| [^15^N_13_]-MeCbl | 679.30 | 149.10 | 20.0 | 47.0 | 28.0 | 100.0 |
|  |  | 361.10 | 36.0 | 28.0 | 26.0 | 100.0 |
|  |  | 982.45 | 20.0 | 30.0 | 38.0 | 100.0 |

**Table S2.** Composition of the minimum defined medium (medium M).

|  | **Components** | **Concentration (g/L)** |
| --- | --- | --- |
| Carbon source | Sodium DL-lactate* | 12 |
| Nitrogen source | (NH_4_)_2_SO_4_ | 3 |
| Minerals | K_2_HPO_4_ | 8.7 |
|  | KH_2_PO_4_ | 6.8 |
|  | MgSO_4_.7H_2_O | 0.2 |
|  | FeSO_4_.7H_2_O | 0.01 |
|  | MnSO_4_.H_2_O | 0.02 |
|  | ZnSO_4_.H_2_O | 0.01 |
|  | NaCl | 0.2 |
|  | CaCl_2_.2H_2_O | 0.132 |
|  | CoCl_2_.6H_2_O | 0.002 |
| Vitamins | Calcium pantothenate | 0.001 |
|  | Biotin | 0.001 |
|  | Thiamin.HCl | 0.001 |
|  | Nicotinic acid | 0.001 |
|  | Riboflavin | 0.001 |
|  | Pyridoxal phosphate | 0.001 |
|  | p-aminobenzoic acid | 0.0002 |
|  | Folic acid | 0.00002 |
| Stimulating agents | Myo-inositol | 0.1 |
|  | Tween 80 | 0.5 |

**20 g of sodium DL-lactate syrup (60%, w/w) was used.*


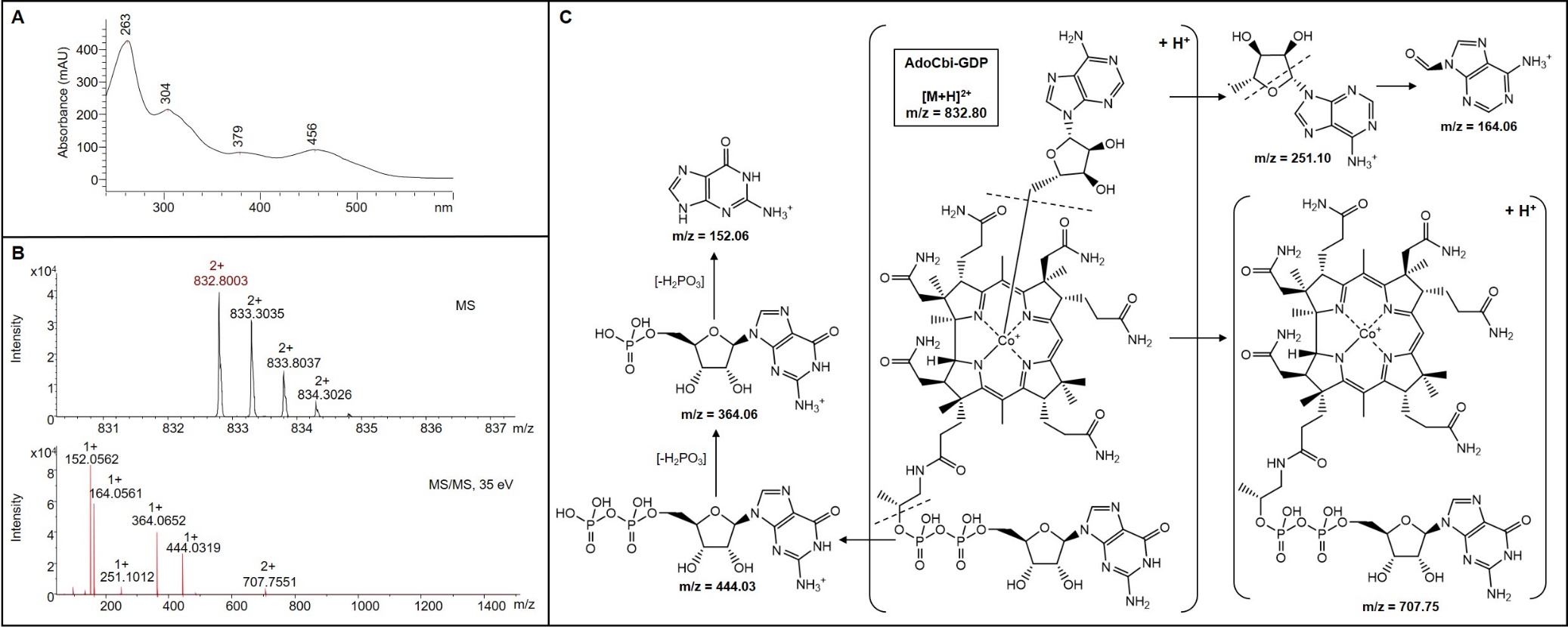


Figure S1. UV-Vis spectrum (A) and UHPLC-QTOF-MS analysis (B) of the peak #2 (AdoCbi-GDP) on Figure 2 with the proposed fragmentation pattern (C). “1+” and “2+” on the MS spectra (B) indicate singly and doubly charged ions, respectively.


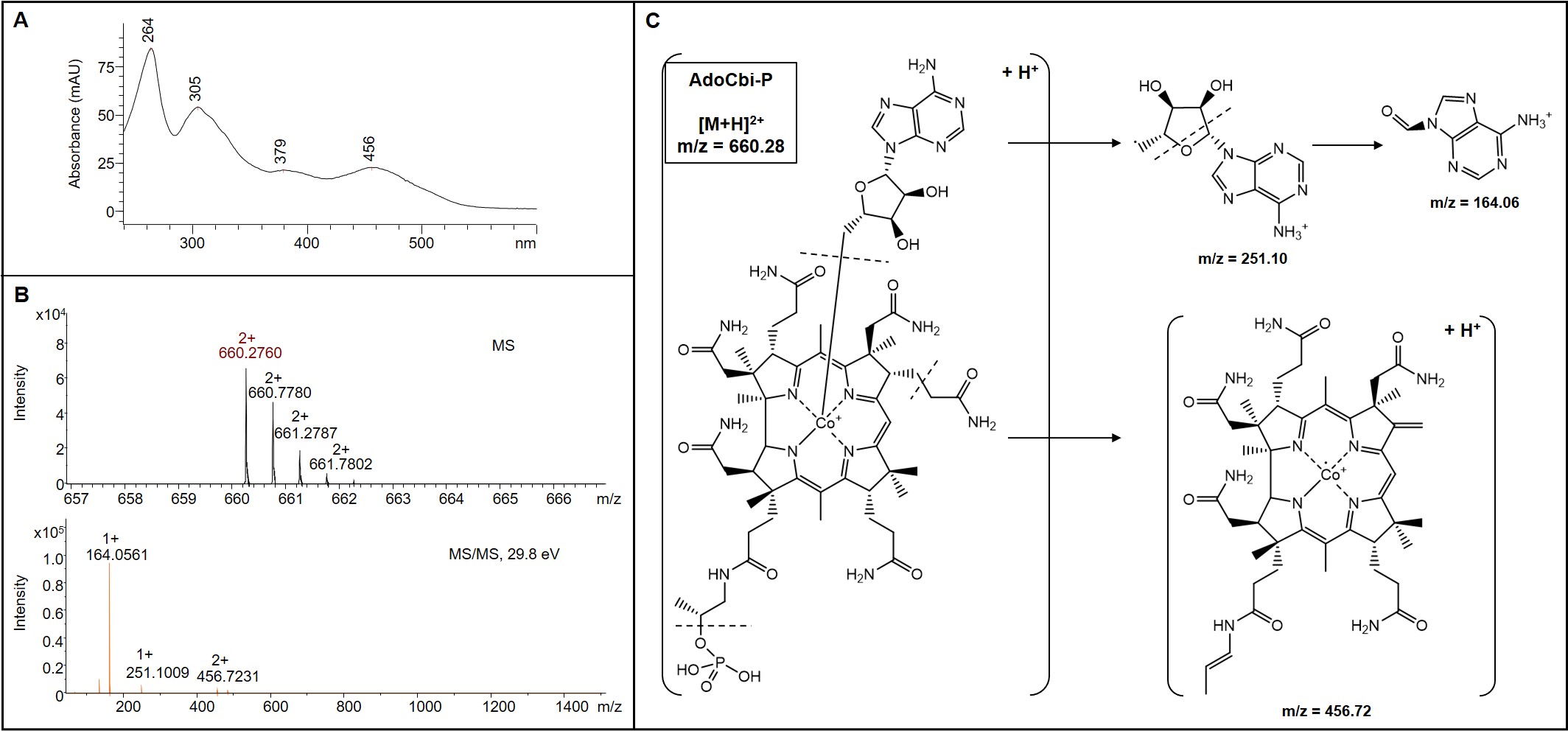


**Figure S2.** UV-Vis spectrum (A) and UHPLC-QTOF-MS analysis (B) of the peak #4 (AdoCbi-P) on Figure 2 with the proposed fragmentation pattern (C). “1+” and “2+” on the MS spectra (B) indicate singly and doubly charged ions, respectively.


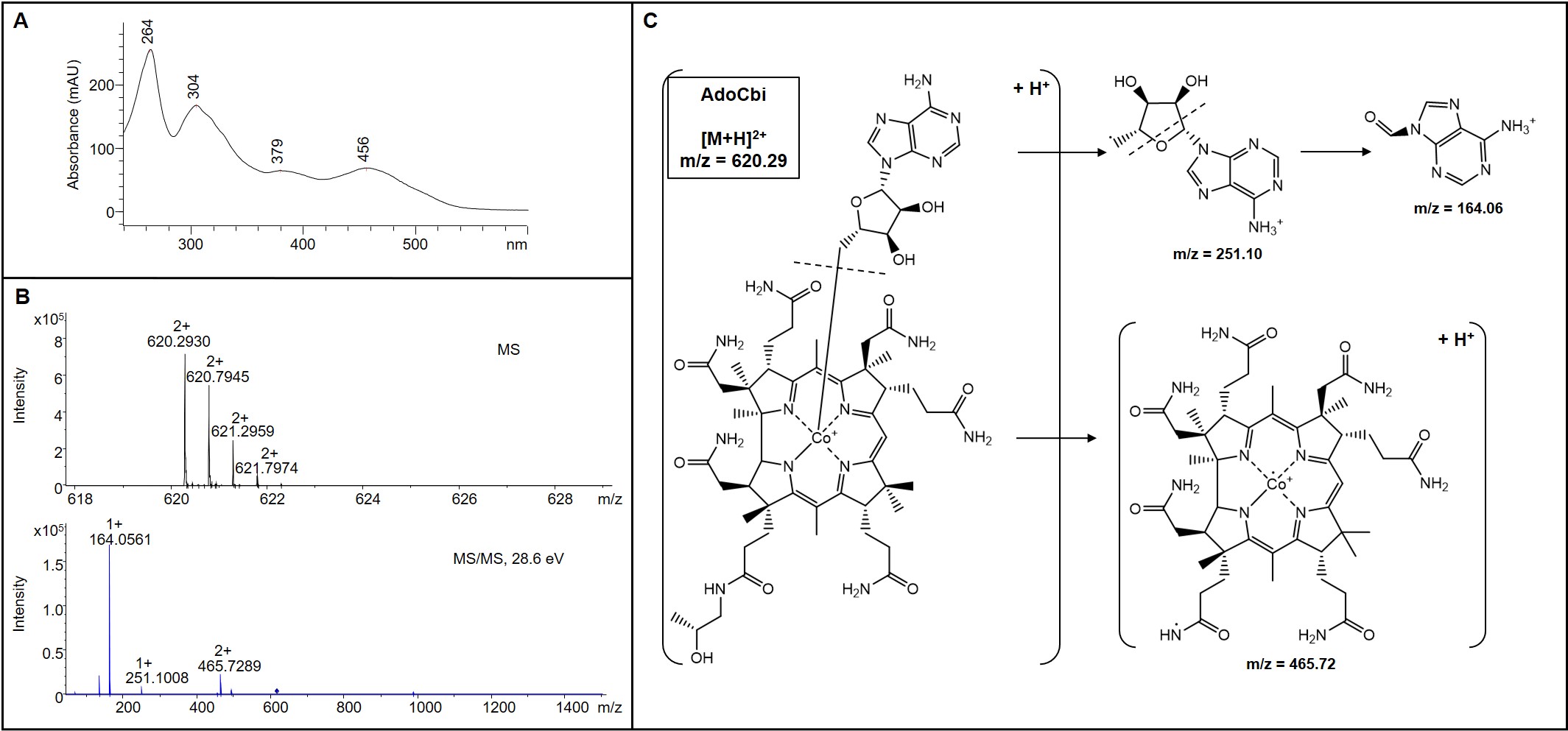


**Figure S3.** UV-Vis spectrum (A) and UHPLC-QTOF-MS analysis (B) of the peak #6 (AdoCbi) on Figure 2 with the proposed fragmentation pattern (C). “1+” and “2+” on the MS spectra (B) indicate singly and doubly charged ions, respectively.


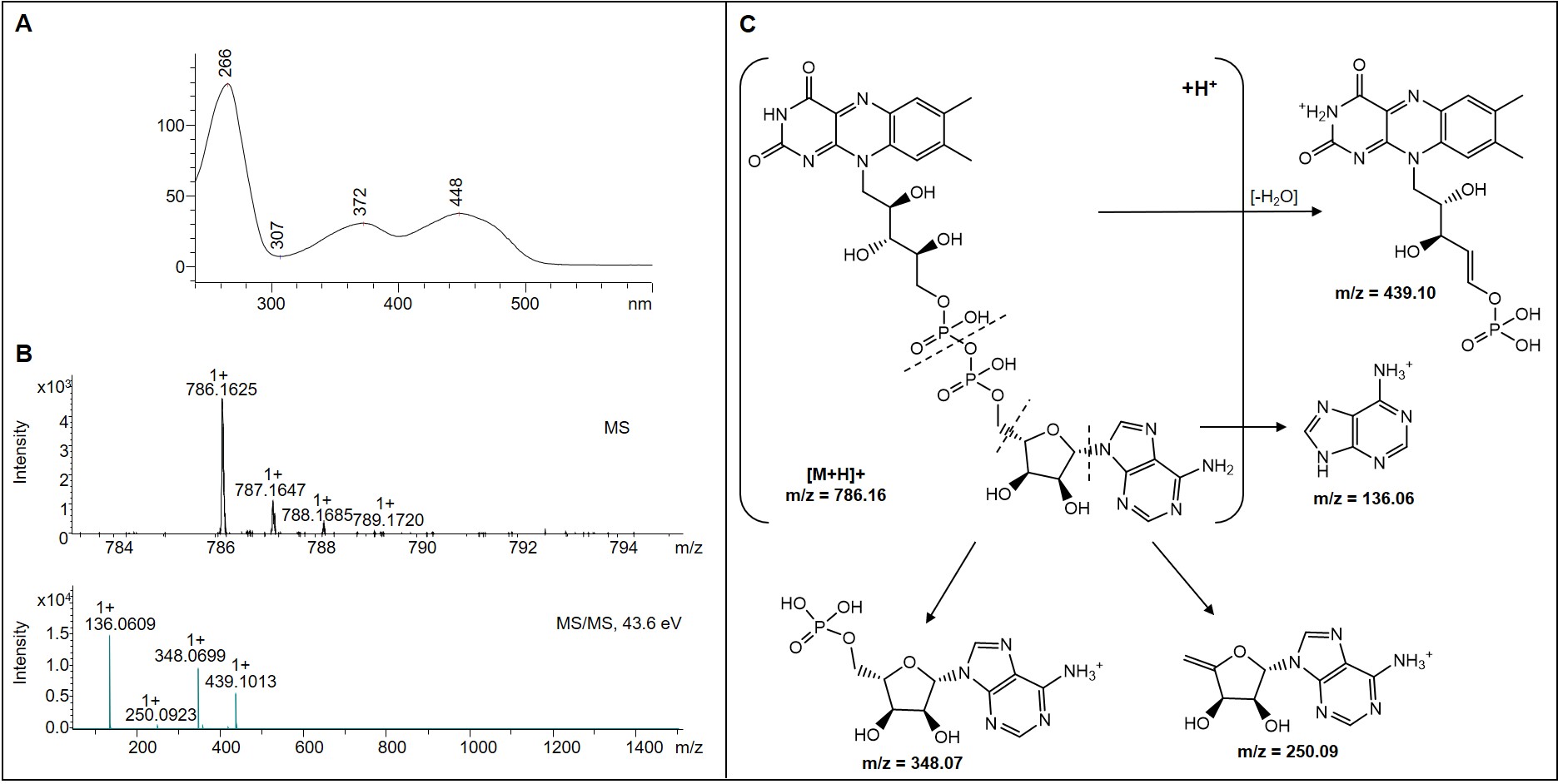


**Figure S4.** UV-Vis spectrum (A) and UHPLC-QTOF-MS analysis (B) of the peak #3 (FAD) on Figure 2 with the proposed fragmentation pattern (C). “1+” on the MS spectra (B) indicates singly charged ions.


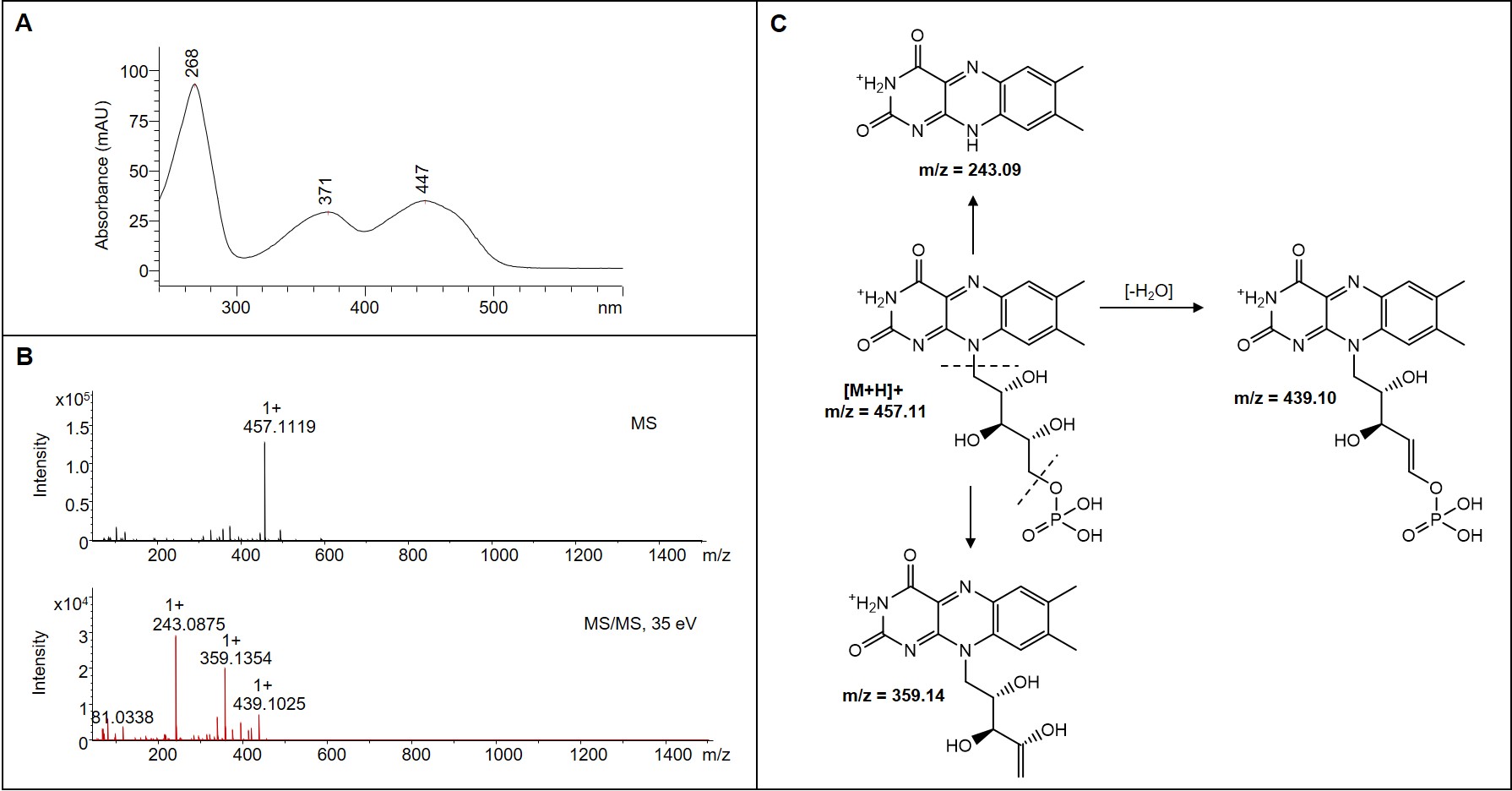


**Figure S5.** UV-Vis spectrum (A) and UHPLC-QTOF-MS analysis (B) of the peak #5 (FMN) on Figure 2 with the proposed fragmentation pattern (C). “1+” on the MS spectra (B) indicates singly charged ions.


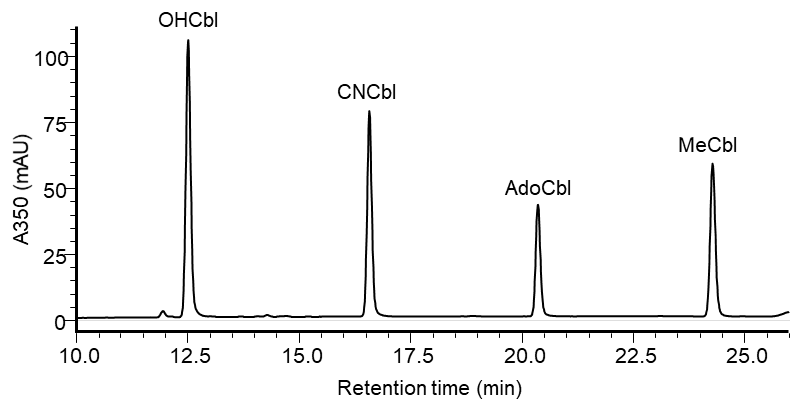


**Figure S6.** HPLC-DAD chromatogram of a standard solution composed of four cobalamin standards under analytical conditions.


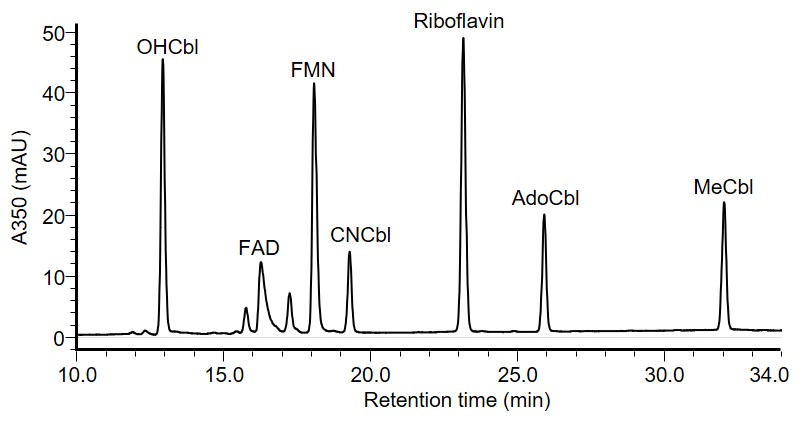


**Figure S7.** HPLC-DAD chromatogram of a standard solution composed of seven standards under semi-preparative conditions.

**Figure S8.** Calibration curve of OHCbl for HPLC-DAD.

**Figure S9.** Calibration curve of AdoCbl for HPLC-DAD.
